# Global payment for health services as a solution in the financial crisis in Europe

**DOI:** 10.5334/ijic.1077

**Published:** 2012-10-31

**Authors:** Guus Schrijvers

**Affiliations:** Julius Centre for Patient Oriented Research, The University Medical Centre Utrecht, Netherlands

**Keywords:** bundled payment, cost control, financial crisis, financing, health services, Integrated Care, payment systems

## Abstract

In these financial difficult years many European governments used global ceilings to control costs of health services. Two scenarios are thinkable. The first is that all individual providers get a budget for their own costs: general practitioners, specialists, hospitals, nursing homes and mental health institutes. The second scenario is to work with global budgets for health care providers servicing a total population. Scientists and policy makers in Europe, North America and Asia need time to design new payment systems based on the idea of global budgeting, bundled payment and shared savings.

## Global payment for health services as a solution in the financial crisis in Europe

In these financial difficult years many European governments used global ceilings to control costs of health services. In the Netherlands the hospitals are not allowed to grow in 2012 by more than 2.5% in volume. In the United Kingdom this percentage is zero. Spain shows a negative percentage. I expect that in the coming years all governments will use this type of macro budgeting for health services and its subsectors as primary care, youth health, hospital care, care for the elderly and mental health services. I expect this because I studied the history of business administration. In 1910, a prosperous period, Taylor introduced his scientific management theory. Based on guidelines of engineers the management of factories designed production lines with pre-calculated norms for processing duration time for the blue collar workers.

I compare the period 2000–2010 with this period in the history. In the last 10 years many European countries introduced targets for length of stay in hospitals, waiting times targets in the accident and emergency departments, health needs norm for admissions to nursing homes and referral standards for general practitioners who refer patients to hospitals or other facilities. This all is known under denominator as disease-related Group (DRG) systems, health care logistics and standardized care pathways. The combination of professional guidelines with integrated digital information systems and routine outcome measurement makes it possible to pre-calculate the length of treatment duration times, the duration of appointments and total length in days of treatment. Based on this knowledge financial and professional agency created quantitative targets for professionals and their institutes.

In the 1920s, the belief in scientific management disappeared. The norms were too high. Workers understood the time measuring engineers and delayed their tempo when the engineers were in the factory. The world famous Hawthorne experiments showed how workers react on management targets. A new idea came up in the Harvard Business School: working with budgets: Don’t give managers professional standards. Give them a lump-sum within which they have to realize their production. A new Law of Boyle became popular: Under budget pressure all fixed standards become fluid. The budget system got a boost in 1929 and the year thereafter, the period of the Great Depression. In the Netherlands a handbook on budgeting was published in 1932 with the motto: a crisis leads to praying and budgeting.

Because of the historical similarities, I expect that European countries will develop budgeting methods for health services. Two scenarios are thinkable. The first is that all individual providers get a budget for their own costs: general practitioners, specialists, hospitals, nursing homes and mental health institutes. Their behavior will be similar as in the past during and after the Great Depression. Putting pressure on the workers (that are now the health professionals), to produce faster and to pay them less. I expect from this scenario under-provision of care, refusing care and cherry picking: selecting easy, cheap patients. Also, an emphasis is also to be expected to reduce the volume of energy costs. I visited a Barcelona hospital using only 50% of their elevators. In Holland I see the abolishment of management tiers within nursing homes under the motto: more hands to the beds. However, the nurses miss leading and innovations disappear. Another reaction within this global budget system is creating waiting times. For instance, less access to a clinic means less pressure on the working professionals working within that clinic. Another option is to refer expensive patients to other providers, for instance teaching hospitals. This is known as patient dump. In this first scenario a lot of mass media scandalizing and policy making based on incidents will show up, making professionals defensive and timid. I give this scenario the name: each for each self.

The second scenario is to work with global budgets for health care providers servicing a total population. For instance, a population of all diabetes patients living in a province or district. In a global budget (for diabetes care for instance) costs are included for GPs, dieticians, medication, internist consultation, eye physician and hospital admission. This is also called ‘bundled payment’. Here, a cost limit exists per patient. However, if more patients show up, then costs are growing. If the target group is not a special patient group but the whole population in a region, the term ‘regional budgeting’ is used. Here the global budgeting is stricter: a fixed amount of money given without considering how many patients show up from the population.

In the UK we see mixed forms of bundled payments and regional budgeting, both being a type of global budgeting. In this country general practitioners purchase a part of the hospital care for their patients. In the United States the Affordable Care Organization (ACO) are to work within global budgeting. However, in the USA a new phenomenon comes up: if the ACO uses less money (with the same quality of care than expected in the allocated bundled payment), it is allowed to spend 50% of the savings within its own mission. This system is known as ‘shared savings’. The second scenario I give the name ‘Together for the patient’.

I prefer the second scenario because it is better for patients, although no empirical evidence exists. Theoretically spoken, in this scenario patients are not dumped by one provider to another because they are too expensive. Within this second scenario I prefer global budgeting with its sub forms of bundled payment and regional budgeting. It depends on the constellation of the health system which of both are most adequate. Not in every health system both sub forms may be applicable. One could think that global regional budgeting might be more easy in systems with regional health authorities as payers, but evidence shows that the USA and Germany have developed some forms as well.

However, I doubt if the second scenario is realizable. At this moment working with global payments is in its infancy. In Germany special payments are applied to disease management programs (DMPs). However, there is a long way to go to bundled payments for all types of costs for chronic patients in DMPs. All right, the Kinzigstal experiment in the South of Germany is popular in the whole of Europe. However, what is successful in a rural area could flop in big cities such as Berlin, Munich, Manchester, Toronto or Utrecht. The Netherlands is also experimenting with bundled payments for patients with a chronic condition as there are persons with diabetes, COPD and heart diseases. Dutch experiments are in preparation for persons with a stroke, neurological conditions such as MS and depression.

Scientists and policy makers in Europe, North America and Asia need time to design new payment systems based on the idea of global budgeting, bundled payment and shared savings. Keep fingers crossed that we have the time.

The International Foundation of Integrated Care tries to bring scientists and policy makers together from everywhere in the world to discuss these ideas on global budgeting and so on. Our next meeting is in Berlin, April 11 and 12, 2013. We are organizing the congress together with the AOK and the Bundesverband Managed Care. Please, save the date.

## About the author

Guus Schrijvers is an economist and professor of public health. He obtained his doctorate in 1980 with a thesis on regionalisation and financing of the English, Swedish and Dutch health care. He was a member of the City Council of Utrecht from 1974 to 1984. In the course of his scientific career, he was one of the architects of integrated new health care systems in the Dutch towns of Almere, Leidsche Rijn and Zwolle. Professor Schrijvers has been head of the Public Health Group at the Julius Centre for Health Sciences and Primary Care of the University Medical Centre in Utrecht since 1987. His work involves research and educational programmes in the field of special groups of health care clients, including heart patients, diabetics, physically disabled cancer patients and the mentally handicapped.

